# The Fate of Altertoxin II During Tomato Processing Steps at a Laboratory Scale

**DOI:** 10.3389/fnut.2019.00092

**Published:** 2019-06-13

**Authors:** Hannes Puntscher, Doris Marko, Benedikt Warth

**Affiliations:** Department of Food Chemistry and Toxicology, Faculty of Chemistry, University of Vienna, Vienna, Austria

**Keywords:** *Alternaria*, emerging contaminants, food safety, liquid chromatography, tandem mass spectrometry, food processing, thermal treatment

## Abstract

Among various agricultural crops, tomatoes are particularly prone to *Alternaria* infections, which are frequently resulting in economic losses and mycotoxin contamination. To investigate potential health concerns implied for consumers, we simulated the storage and food processing steps of intact and blended tomatoes after addition of the highly genotoxic secondary metabolite altertoxin II. We observed a significant decrease in altertoxin II concentrations in samples stored at room temperature and particularly those undergoing thermal treatment by employing a validated LC-MS/MS method. When kept at room temperature, 87–90% of ATX-II was recovered after 1.5 h in raw tomato purees and purees heated before ATX-II addition, and 47–49% were recovered after 24 h. In intact tomato fruits the recovery was 23% after 1.5 h and <1% after 24 h. In heated purees (100°C for 30 min after ATX-II addition), also only minor concentrations accounting for 2-4% were determined. Moreover, the reduction of the compound's epoxide group to the alcohol, i.e., the formation of altertoxin I was demonstrated in intact tomato fruits (7–12%), suggesting enzymatic biotransformation of the xenobiotic by the plant's metabolism.

## Introduction

**Figure 1:**
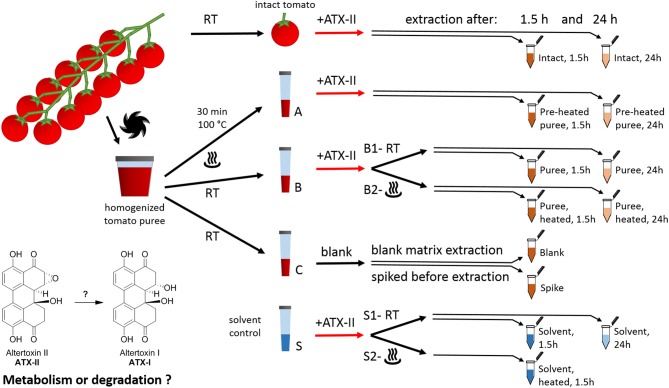
Study design for investigating the fate of altertoxin II in tomato commodities taking into account mechanical and thermal processing steps.

Altertoxin II (ATX-II) is a toxic secondary metabolite produced by the fungal genus *Alternaria* (Figure 1). *Alternaria* spp. are ubiquitous saprophytes and plant pathogens, often responsible for considerable economic losses due to infections of agricultural crops like cereals, tomatoes, and oil seeds (1–5). Moreover, health risks might be implied for consumers by related mycotoxin contamination. The ability of proliferation even at lower temperatures allows for post-harvest infections during refrigerated storage and transport (5, 6). However, temperature and humidity influence *Alternaria* growth and toxin production (7). The European Food Safety Authority released a dietary exposure assessment evaluating the four *Alternaria* toxins alternariol (AOH), alternariol monomethyl ether (AME), tentoxin (TEN), tenuazonic acid (TeA). For the genotoxic AOH and AME, estimated exposure levels indicated a possible health concern (1, 8). These toxins were also shown to be chemically relatively stable during food processing (9, 10).

ATX-II is significantly more genotoxic than AOH (11–13), however, it has not been reported in naturally contaminated food so far. Due to the lack of commercially available reference material, it is not included in standard assays. Only a few LC-MS based methods allow to determine and accurately quantify this potent toxin, after having isolated the compound from fungal cultures (14–17). While genotoxic and mutagenic effects of AOH described *in vitro* (18) were linked to topoisomerase I and II poisoning (19), the mechanism of action related to ATX-II has not been elucidated so far. The reactive epoxide of ATX-II is likely to be involved in toxicological effects. However, even altertoxin I (ATX-I, Figure 1), structurally the same scaffold but lacking the epoxide, was reported to be mutagenic to a certain extent *in vitro* (20). While ATX-II did not show estrogenic effects in Ishikawa cells (21), chemical degradation of the compound was suggested in the presence of the anthocyanin delphinidin (22). In several cell lines (Caco-2, HCT 116, HepG2, and V79), it has been reported that the epoxide of ATX-II was reduced to an alcohol resulting in ATX-I, which seemed not to be further metabolized in Caco-2 cells (23, 24).

In this study, we investigated the fate of ATX-II in intact and homogenized tomato fruits during food processing steps at a laboratory scale. Given that tomatoes are frequently infected by the ubiquitous plant pathogens *Alternaria* spp., the stability and persistence of this highly genotoxic compound are of general interest. ATX-II contamination might be a relevant, yet under-investigated health issue for consumers.

## Materials and Methods

### Reagents, Solvents, and Chemicals

ATX-II was isolated in-house from *Alternaria alternata* cultures grown on rice (25), and confirmed by NMR. ATX-I was purchased from Romer Labs (Tulln, Austria). Methanol (MeOH), water, and acetonitrile (ACN) were purchased from Honeywell (Seelze, Germany), and ammonia solution (25% in water), ammonium acetate (all for LC-MS), MeOH (HPLC grade), and acetic acid (p.a.) from Sigma Aldrich (Steinheim, Germany).

ATX-II was dissolved in MeOH (250 μg/mL) and further diluted in the same solvent (25 μg/mL). A multi-component calibration solution (including ATX-I, as well as alterperylenol (ALP) and stemphyltoxin-III (STTX-III), both isolated from rice cultures) was used for external calibration of additional *Alternaria* toxins. All solutions were demonstrated to be stable during storage at −20°C and repeated measurements at 10°C over 72 h.

### Sample Preparation

Cherry tomatoes were purchased from a retail market in Vienna, Austria, in May 2018. The study design is presented in Figure 1. All experiments were performed in triplicates. Firstly, 12 fruits from the same truss without visible fungal infections were rinsed with water. Six randomly picked tomatoes (“intact tomato” samples) were stored at room temperature until the start of the experiment (i.e., the addition of the toxin). The remaining six tomatoes were cut into pieces and homogenized using a FastPrep-24 5G™ High Speed Homogenizer (MP Biomedicals Life Sciences, USA). Aliquots of the resulting tomato puree (1 g each) were transferred to plastic tubes (15 mL, Sarstedt, samples A, B, and C). Six of these were heated at 100°C for 30 min (“Pre-heated” puree samples, samples A) using a water bath. Samples B and C were kept at room temperature in the meantime. As a solvent control, nine tubes were filled with 1 mL Milli-Q water (“solvent control,” samples S).

ATX-II stock solution (250 μL/mL) was injected into six “intact tomato” fruits (35–45 μL per tomato fruit). Therefore, a pipette tip was used to pierce the peel once and inject the solution into the fruit (about 10–15 mm underneath the peel). For the samples A, B, and S, the ATX-II working solution (25 μg/mL) was used. All ATX-II additions were adjusted to result in a final concentration of 1 μg ATX-II per 1 g of each sample. No ATX-II solution was added to the samples C at this point. Three of these were providing for “blank” matrix samples to investigate potential natural contamination. The other three samples C were spiked after 1.5 h, right before extraction (“Spike” samples), providing concentrations at the formal time point 0 h (considered as 100%). All puree and solvent samples were vortexed gently after the addition of ATX-II to allow for appropriate homogenization. Subsequently, six samples B (B2 in Figure 1) and three samples S (S2 in Figure 1) were heated to 100°C for 30 min, mimicking a thermal processing step. Three “intact tomato” fruits, three samples A (“pre-heated” puree), three samples B1 (“non-heated” puree), three samples B2 (“heated” after ATX-II addition), six samples C (for blank and spiking), and six samples S (“non-heated” S1, “heated” S2) were extracted after 1.5 h. The remaining samples were extracted after 24 h. The first time point was chosen to allow comparison with data in literature (22, 24), while the second was to investigate subsequent progress and trends.

### Sample Extraction

All samples were extracted as described in Puntscher et al. (16). Intact tomato fruits were chopped and homogenized before applying the same procedure as for the preparation of the puree samples (see above). All homogenized samples (1.000 ± 0.005 g) were extracted by adding solvent (5 mL, methanol/water/acetic acid, 79/20/1, v/v/v) and shaking for 60 min using an over-head shaker (Roto-Shake Genie, Scientific Industries, USA). These extracts were subsequently diluted 1:1 with methanol/water (10/90, v/v), centrifuged (20.000 rcf, 4°C, 15 min) and stored at −20°C until LC-MS/MS measurement.

### Mass Spectrometric Quantitation

LC-MS/MS analysis was performed on a high-performance liquid chromatography (UHPLC) system (UltiMate3000) coupled to a triple-quadrupole mass spectrometer (TSQ Vantage, Thermo Scientific) applying a validated method (16) and Tracefinder^TM^ (version 3.3) software for data evaluation. Briefly, the mass spectrometric system was operated in multiple reaction monitoring mode (MRM, quantifier/qualifier ions for ATX-II: *m/z* 349 -> 285/331, and for ATX-I *m/z* 351 -> 315/333) using negative electrospray ionization. Both altertoxins were quantified by matrix-matched calibration (ATX-II: 0.1–100 ng/mL, ATX-I: 0.2–200 ng/mL). For quality control, standards in pure solvent were included in the same sequence (10% methanol in water).

## Results and Discussion

**Figure 2:**
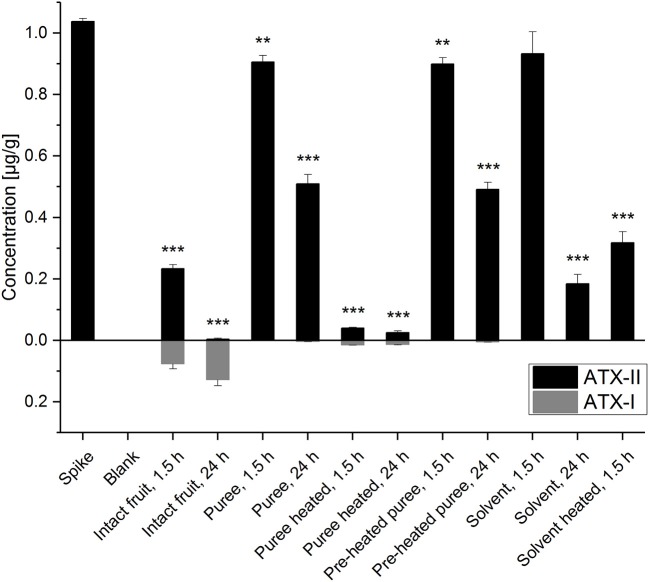
Altertoxin I and II concentrations in the experimental tomato and solvent control samples. Significant differences between the concentrations in the test samples and the “spike” samples were calculated applying an unpaired heteroscedastic Student's *t*-tests and are indicated by **p < 0.01 and ***p < 0.001.

The detection and quantitation of ATX-II and ATX-I was conducted by LC-MS/MS analysis (Figure 2 and Table S1). The performed spiking experiment confirmed a satisfying extraction efficiency of 102-104% for ATX-II in the tomato matrix. Natural contamination of *Alternaria* toxins was excluded by the analysis of blank matrix extractions. ATX-II concentrations were decreasing in all samples over time. Surprisingly, after 1.5 h at room temperature, ATX-II levels were reduced very similarly to 87-90% (Figure 2) in both tomato puree types, e.g., the pre-heated samples A and the non-heated samples B1, as well as in the “solvent control” samples S1. The comparable decrease in the solvent control samples suggests a generally limited chemical stability or similar reactivity of ATX-II at room temperature *per se*. This has also been reported by Zwickel et al. (17). After 24 h at room temperature, the levels further declined to 47-49% in the tomato puree samples (A and B1) and to 18% in S1. This indicates that the polar solvent water is not favorable for stable conditions. ATX-II seemed to degrade/react slower in tomato matrix, potentially related to stabilizing pH conditions or matrix-related interactions.

Despite the fact that thermal stress generally reduces enzymatic activity, ATX-II concentrations for the thermally treated (“pre-heated”) puree (A) and the non-heated puree (B1) were almost identical after 1.5 and 24 h, respectively. This raises the question, whether xenobiotic metabolism was reduced also by the applied mechanical stress during homogenization. Short blending steps of 40 s were intended to minimize thermal stress applied to the matrix. However, the disruption of the tomato tissue and cell structures might have inactivated enzymes to some extent. Thermal treatment of 30 min at 100°C for the samples B2 (after ATX-II addition) clearly led to the most efficient reduction of ATX-II (>95%) indicating enhanced/accelerated reactivities. Only 4 and 2.5% of the added ATX-II were recovered after 1.5 and 24 h, respectively. Comparably higher ATX-II levels (31%) were determined in the heated solvent control samples (S2) after 1.5 h. Hence, ATX-II decrease may be related to matrix interactions allowing for adsorption effects or covalent binding. Finally, ATX-II levels in intact tomato fruits were reduced to 23% after 1.5 h and therefore much more efficiently as in all other samples at room temperature. After 24 h, <1% of the added amount was recovered. This strongly indicates active plant metabolism as an effective tool to deal with the xenobiotic ATX-II. Interestingly, 7% of ATX-II was recovered as ATX-I after 1.5 h and 12% after 24 h (for these calculations the molar masses of the compounds were taken into account). The tomato tissue seems capable of reducing the epoxide group of ATX-II to the corresponding hydroxyl-group of ATX-I. This metabolic pathway has already been reported in *in vitro* experiments in human and animal cell lines (23, 24), but not in plants. De-epoxidation is known as a detoxification process for other epoxide-holding mycotoxins, including the trichothecene deoxynivalenol (DON) (26, 27). Mammalian epoxide hydrolases were reported to play a major role in converting a large number of structurally different epoxides to the corresponding less reactive vicinal diols and are therefore considered as important detoxification enzymes (28). In a recent *in vivo* study, a complex *Alternaria* culture extract containing high concentrations of ATX-I and ATX-II (among other *Alternaria* toxins) was administered to rats *via* gavage. ATX-I, but not ATX-II, was recovered in both urine and fecal samples (29).

In the presented study, much smaller ATX-I amounts were also determined in other tomato samples, but not in the solvent controls. As observed for ATX-II, ATX-I concentrations were nearly the same for the puree samples A and B1 (0.7–0.9 ng/g after 1.5 h, 3.1–5.0 ng/g after 24 h, corresponding to <0.1 and 0.3% of the added ATX-II). Heating of samples after ATX-II addition led to slightly higher ATX-I concentrations (13.4–14.7 ng/g, corresponding to 1.3–1.4% of the added ATX-II). Tomato matrix components appear to catalyze chemical reduction of the ATX-II epoxide. However, the conversion reaction in intact tomatoes is far more efficient (by a factor of up to 100). Since not 100% of the decreased ATX-II was converted to ATX-I, further decomposing products might be identified in the future, as neither alterperylenol, nor stemphyltoxin-III was determined in any sample.

## Conclusion and Outlook

We demonstrated that the concentrations of the highly genotoxic ATX-II added to tomato products were decreased when mimicking food processing at a laboratory scale. Already at room temperature, this *Alternaria* toxin was of limited stability in both, tomato puree and the solvent control water. No notable difference was determined between tomato purees, which were heated before ATX-II addition and those that were not. By thermal treatment of the contaminated puree, ATX-II levels were significantly reduced, indicating increased matrix-related reactivity, possible adsorption, covalent binding, chemical modification, and/or degradation. Intriguingly, intact tomato fruits demonstrated a more efficient reduction with <1% ATX-II recovered after 24 h at room temperature. The conversion of ATX-II to ATX-I by de-epoxidation (up to 12% after 24 h) suggests effective plant detoxification. Potential health concerns caused by ATX-II or its degradation/reaction products cannot be excluded. Future studies should investigate the structure(s) of the latter as well as their toxicological potential. Moreover, large-scale food surveys are required to investigate the occurrence of perylene quinones in food commodities.

## Author Contributions

HP, DM, and BW designed the study. HP planned and carried out the experiments, analyzed the data, and took the lead in writing the manuscript. All authors discussed the results, provided critical feedback, and contributed to the final manuscript. BW supervised the project.

### Conflict of Interest Statement

The authors declare that the research was conducted in the absence of any commercial or financial relationships that could be construed as a potential conflict of interest.

## Abbreviations

ALP: alterperylenol
AME: alternariol monomethyl ether
AOH: alternariol
ATX-I: altertoxin I
ATX-II: altertoxin II
LC: liquid chromatography
MRM: multiple reaction monitoring
MS: mass spectrometry
STTX-III: stemphyltoxin III
TeA: tenuazonic acid
TEN: tentoxin.
