# Supplementary material for: The Fate of Altertoxin II During Tomato Processing Steps at a Laboratory Scale
Source: Front Nutr. 2019 Jun 13;6:92. doi: 10.3389/fnut.2019.00092 (PMC6584911; doi:10.3389/fnut.2019.00092)
Supplement: Supplementary file 1 [file Table_1.DOCX]

**Electronic supplementary information**

**The fate of altertoxin II during tomato processing steps**

**at a laboratory scale**

Hannes Puntscher^1^, Doris Marko^1^, Benedikt Warth^1^

^1^Department of Food Chemistry and Toxicology, Faculty of Chemistry, University of Vienna, Währingerstr. 38, 1090 Vienna, Austria

**CORRESPONDING AUTHOR:** Benedikt Warth, University of Vienna, Department of Food Chemistry and Toxicology, Währingerstr. 38, 1090 Vienna, Austria.

Phone: +43 1 4277 70806

E-mail: [benedikt.warth@univie.ac.at](mailto:benedikt.warth@univie.ac.at)

ORCID: <https://orcid.org/0000-0002-6104-0706>

**Table S1** Sample overview listing all ATX-II and ATX-I concentrations

|  |  | **Time points** | | | | | |  | | **Heating** | | |  | **ATX-II** | | **ATX-I** | |
| --- | --- | --- | --- | --- | --- | --- | --- | --- | --- | --- | --- | --- | --- | --- | --- | --- | --- |
|  |  | **0 h** | | **1.5 h** | | **24 h** | |  | | **Pre-addition** | | **Post-addition** |  | **[µg/g]** | **[%]** | **[µg/g]** | **[%]** |
|  |  |  | |  | |  | |  | |  | |  |  |  |  |  |  |
| **Sample C (Spike)** |  | x | |  | |  | |  | |  | |  |  | 1.038 ± 0.009 | 100 % | 0 | 0 % |
| **Sample C (Blank)** |  |  | |  | |  | |  | |  | |  |  | 0 | 0 % | 0 | 0 % |
|  |  |  | |  | |  | |  | |  | |  |  |  |  |  |  |
| **Intact tomato fruits** |  |  | | x | |  | |  | |  | |  |  | 0.234 ± 0.013 | 23 % | 0.077 ± 0.016 | 7 % |
|  |  |  | |  | | x | |  | |  | |  |  | 0.004 ± 0.004 | 0.4 % | 0.127 ± 0.02 | 12 % |
|  |  |  | |  | |  | |  | |  | |  |  |  |  |  |  |
| **Samples B1 (non-heated)** |  | |  | | x | |  | |  | |  |  |  | 0.906 ± 0.021 | 87 % | 0.001 ± 0 | 0.1 % |
|  |  |  | |  | | x | |  | |  | |  |  | 0.510 ± 0.030 | 49 % | 0.003 ± 0.001 | 0.3 % |
| **Samples B2 (heated)** |  |  | | x | |  | |  | |  | | x |  | 0.040 ± 0.002 | 4 % | 0.015 ± 0.001 | 1.4 % |
|  |  |  | |  | | x | |  | |  | | x |  | 0.025 ± 0.006 | 2 % | 0.013 ± 0.001 | 1.3 % |
|  |  |  | |  | |  | |  | |  | |  |  |  |  |  |  |
| **Samples A (pre-heated)** |  |  | | x | |  | |  | | x | |  |  | 0.899 ± 0.021 | 87 % | 0.001 ± 0 | 0.1 % |
|  |  |  | |  | | x | |  | | x | |  |  | 0.491 ± 0.023 | 47 % | 0.005 ± 0.001 | 0.5 % |
|  |  |  | |  | |  | |  | |  | |  |  |  |  |  |  |
| **Solvent control (S1, 1.5 h)** |  |  | | x | |  | |  | |  | |  |  | 0.933 ± 0.071 | 90 % | 0 | 0 % |
| **(S1, 24 h)** |  |  | |  | | x | |  | |  | |  |  | 0.184 ± 0.031 | 18 % | 0 | 0 % |
| **(S2, 24 h)** |  |  | | x | |  | |  | |  | | x |  | 0.318 ± 0.036 | 31 % | 0 | 0 % |
|  |  |  | |  | |  | |  | |  | |  |  |  |  |  |  |
